# Extraoral low-level laser therapy can decrease pain but not edema and trismus after surgical extraction of impacted mandibular third molars: a randomized, placebo-controlled clinical trial

**DOI:** 10.1186/s12903-022-02461-2

**Published:** 2022-09-20

**Authors:** Ehsan Momeni, Farahnaz Kazemi, Parisa Sanaei-rad

**Affiliations:** 1 Deparment of Oral Medicine, School of Dentistry, Arak University of Medical Sciences, Arak, Iran; 2 Deparment of Endodontics, School of Dentistry, Arak University of Medical Sciences, Arak, Iran

**Keywords:** Edema, Low level light therapy, Molar, Third, Pain, Trismus, Tooth, Impacted

## Abstract

**Background:**

This study aimed to assess the effect of extraoral 940 nm low-level diode laser on pain, edema, and trismus following surgical extraction of impacted mandibular third molars.

**Materials and methods:**

This split-mouth, randomized, placebo-controlled clinical trial evaluated 25 patients with bilaterally impacted mandibular third molars. One side of the jaw was randomly assigned to the laser and the other side to the control group. The laser quadrant received 940 nm diode laser irradiation (0.5 W, 10 J/cm^2^, continuous-wave mode, 20 s) at three points in the master muscle in contact mode immediately after surgical extraction of third molar. The third molar in the placebo quadrant was extracted after 2 weeks by the same surgeon using the same standard approach. The pain score was measured at 2 and 7 days postoperatively using a visual analog scale (VAS). To assess trismus, the distance between the incisal edges of the upper and lower central incisors was measured in maximum opening. To assess edema, the distance between the tragus and chin point was measured before and immediately after surgery and after 2 and 7 days. Data were analyzed using t-test, ANOVA, and Bonferroni test.

**Results:**

The mean pain score in the first 7 days was significantly lower in the laser group (*P* < 0.05). Edema and trismus were the same in both groups (*P* > 0.05). Number of analgesics taken was significantly lower in the laser group (*P* < 0.05).

**Conclusion:**

Single-session irradiation of 940 nm diode laser can effectively decrease pain following third molar extraction surgery.

*Trial registration number*: IRCT20141209020258N91 on 29/12/2018.

## Introduction

Surgical extraction of impacted mandibular third molars is a common surgical procedure, which is often associated with postoperative complications such as pain, edema, and trismus due to intraoperative trauma [1, 2]. The pain usually aggravates in the first 5 h postoperatively, and then gradually decreases until the end of the first week [3].

Several strategies have been suggested to minimize postoperative complications, such as the systemic use of corticosteroids, enzymes, or non-steroidal anti-inflammatory medications, surgical closure techniques with/without placement of a drain, use of ice packs, cryotherapy, and laser therapy [4–9]. Considering the side effects of analgesics and relative inefficacy of some other suggested strategies, laser therapy seems to be a novel modality for this purpose [10, 11].

Low level lasers have several applications in dentistry, and are used for enhancement of wound healing, acceleration of tissue regeneration, gingival depigmentation, treatment of temporomandibular disorders, alleviation of chronic orofacial pain, and induction of bone regeneration [12]. The anti-inflammatory mechanism of low-level laser therapy (LLLT) is based on inhibition of IL-6, IL-10, tumor necrosis factor-alpha, and monocyte chemotactic protein-1 [13]. Evidence shows that diode laser in 940 to 980 nm wavelengths is effective for reduction of postoperative inflammation, enhancement of wound healing, and acceleration of tissue regeneration.

The efficacy of LLLT for pain relief following surgical extraction of third molars is still a controversial topic. Limited number of studies have reported the optimal clinical efficacy of LLLT for reduction of postoperative pain, edema, and trismus [14–18] while some others have denied the optimal efficacy of laser for this purpose [19–21]. Also, most previous studies reporting the optimal efficacy of LLLT have tried multiple sessions while new generations of lasers can be used in one single session. However, studies on the efficacy of single-session LLLT for pain relief are limited [22–24]. In a study by landlucci et al., LLLT was performed to study the reduction of pain, swelling, and trismus following the surgical extraction of third molars. The authors used a combination of intra- and extraoral protocol and demonstrated that all associated discomforts were reduced [24]. In a recent study, Hamzah et al. investigated the effect of intraoral single-session LLLT on post-operative pain and showed significant pain decrease after tooth extraction [25]. In a similar study, Hamid et al., evaluated the intraoral LLLT on postoperative pain following mandibular third molar surgery and demonstrated effective pain reduction using 810-nm GaALAs laser [26]. According to our knowledge, there is no study investigated the effect of single-session extraoral LLLT on the amount of pain, swelling and trismus after molar extraction. Thus, this study aimed to assess the effect of extraoral 940 nm low-level diode laser on pain, edema and trismus following surgical extraction of impacted mandibular third molars.

## Materials and methods

### Trial design

This split-mouth, randomized, placebo-controlled clinical trial evaluated 25 healthy patients over 18 years of age (range 18–40 years) with asymptomatic, bilaterally impacted, mandibular third molars. The study was approved by the National Committee for Ethics in Biomedical Research and registered in the Iranian Registry of Clinical Trials **(**IRCT20141209020258N91) on 29/12/2018**.** The patients signed informed consent forms prior to participation in the study.

Sample size was calculated considering alpha = 0.05, beta = 0.2 and study power of 80%.

The inclusion criteria were: ASA class 1 patients, no medication intake for 1 week prior to the surgical procedure, no allergy to anesthetic agents, no systemic diseases, no chronic pain, no intake of anti-inflammatory medications or analgesics for 2 weeks prior to the study, no intake of corticosteroids, absence of pericoronitis, no smoking, and no pregnancy or lactation. Also, in each patient, the bilaterally impacted mandibular third molars had to be in the same class according to the Winter’s classification (mesioangular, distoangular, vertical, or horizontal) [27], Pell and Gregory classification (relationship of tooth with the anterior border of ramus) [28], and Gregory A, B, C classification (determining the level of difficulty of the procedure by comparing the occlusal height of the third molar relative to that of the second molar) [28]. Also, surgical extraction of all teeth required bone removal and sectioning of the teeth.

The exclusion criteria were: unwillingness for participation in the study, postoperative pharmaceutical complications and side effects, allergy to the used materials, postoperative infection or acute abscess, not showing up for the second surgery after 2 weeks, not consenting to laser therapy after surgery, and not completing the questionnaire after surgery.

### Randomization process

Considering the randomized split-mouth design of the study, the laser side was determined by flipping a coin in each patient. The contralateral quadrant served as the placebo group.

### Intervention

**Fig. 1:**
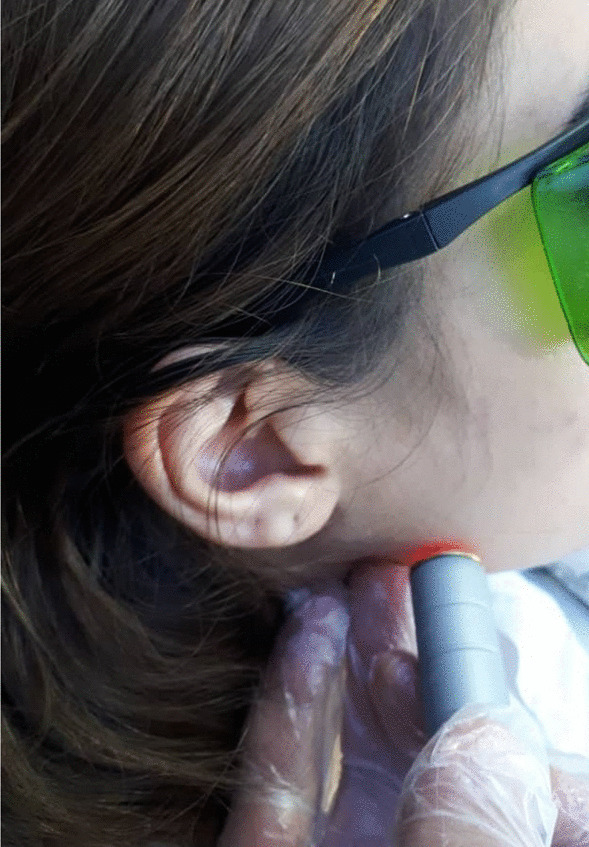
Laser irradiation

The patients received inferior alveolar nerve block, lingual nerve block, and buccal nerve block using two 1.8 mL cartridges of 2% lidocaine plus 1:80.000 epinephrine (Colombia, Guarne, New stetic S.A). An incision was made using a #15 scalpel (Bromed, Ontario, USA) and a mucoperiosteal flap was elevated using an elevator (Nordent, IL, USA). Bone was removed from the buccal and distal areas using a #8 round carbide bur (Dentsply, Ballaigues, Switzerland) and a low-speed hand-piece (NSK, Tokyo, Japan). The impacted tooth was sectioned into pieces by a 703 fissure bur (Dentsply, Ballaigues, Switzerland) and a low-speed handpiece under saline irrigation and removed. The surgical wound was closed by non-absorbable 3–0 silk sutures using a 3.8 reverse cutting needle (SMI, Steinerberg, Belgium). After termination of the surgical procedure of the laser side and immediately after suturing, the patients received 940 nm extraoral diode laser irradiation (Epic) with 0.5 W/cm^2^ power, maximum output power of 2.75 W, and spot size of 8 × 35 mm. An oral medicine specialist laser-irradiated the surgical site while the placebo side received no laser irradiation. The fiber tip was in contact with the skin and laser was irradiated to a triangular area hypothetically formed between the point of insertion of the masseter muscle at the angle of mandible, the tragus lobe of the ear, and the mesial side of the mandibular second molar (Fig. 1). The total duration of radiation was 60 s, and laser was irradiated with a total energy of 10 J/cm^2^ to each point. The surgical extraction of impacted mandibular third molar in the placebo side was performed after 2 weeks. In the placebo side, laser probe was positioned over the area while the laser was in off mode. Thus, the patients were blinded to the group allocation of their mandibular quadrants.

After surgery, the patients were prescribed 500 mg amoxicillin every 8 h for 7 days, 400 mg ibuprofen every 12 h for 3 days, and chlorhexidine mouthwash for 7 days.

Presence/absence of edema was determined by measuring the distance between the chin point and the inferior border of the external auditory canal as described by Markovic and Todorovic [29].

Trismus was determined by measuring the distance between the incisal edges of the upper and lower right central incisors in maximum opening using a calibrated digital caliper before and immediately after surgery and also after 2 and 7 days.

Pain was determined using a visual analog scale (VAS). The patients were requested to express the level of pain experienced using a 10-cm VAS; 0 indicated no pain while 10 indicated most severe pain imaginable. To enhance the patients’ understanding of the VAS scores, the Faces pain scale was also used in addition to the linear VAS. A questionnaire was designed for this purpose and the patients were requested to record their pain score for 7 days, postoperatively. They were also asked to record the number of analgesics taken during this time period.

Data were analyzed using SPSS version 23 (SPSS Inc., IL, USA). The measures of central dispersion were reported for descriptive variables. Comparisons were made using independent t-test, paired t-test, ANOVA, and Bonferroni test. Level of significance was set at 0.05.

## Results

A total of 25 patients with a mean age of 26.84 ± 3.61 years were evaluated, including 14 females (56%) and 11 (44%) males.

### Number of analgesics taken

The mean number of analgesics taken was significantly lower in the laser group (5.4 ± 2.98 versus 11.08 ± 5.18, *P* = 0.00).

### Edema

**Fig. 2:**
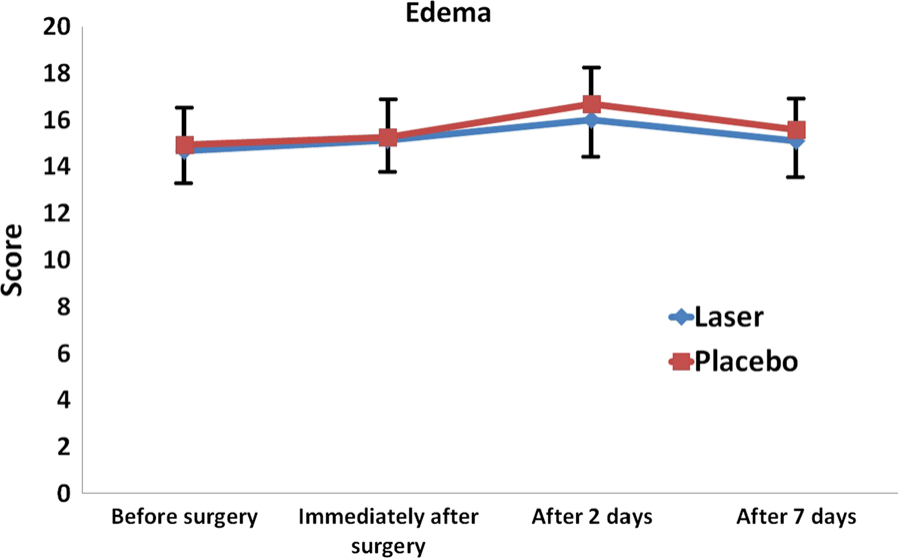
Mean score of edema in different time points

Figure 2 shows the mean score of edema in the two groups at different time points. Edema was not significantly different in the two groups at any time point (*P* > 0.05). Repeated measures ANOVA revealed that the mean score of edema increased within each group in the first 2 days and then decreased; this trend of change was statistically significant in both groups (Table 1, *P* < 0.05). However, the two groups were not significantly different in this regard; although the increase in edema was slightly greater in the first 2 days in the control group (*P* = 0.258).

**Table 1:** Trend of change in edema at different time points in the two groups

Time (I)	Time (J)	Laser			Placebo		
		Mean difference	Std. deviation	*P* value	Mean difference	Std. deviation	*P* value
Before surgery	Immediately after surgery	− 0.444^*^	0.079	0.0001	− 0.328^*^	0.061	0.0001
	After 2 days	− 1.300^*^	0.103	0.0001	− 1.740^*^	0.150	0.0001
	After 7 days	− 0.416^*^	0.105	0.003	− 0.628^*^	0.097	0.0001
Immediately after surgery	After 2 days	− 0.856^*^	0.134	0.0001	− 1.412^*^	0.142	0.0001
	After 7 days	0.028	0.136	0.999	− 0.300^*^	0.092	0.020
After 2 days	After 7 days	0.884^*^	0.095	0.0001	1.112^*^	0.117	0.0001

* *P* < 0.05

### Mouth opening

**Fig. 3:**
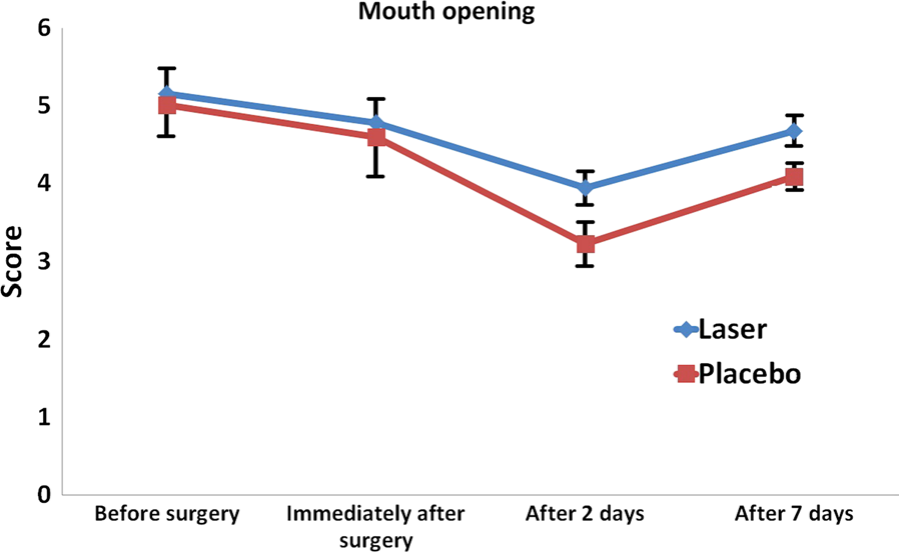
Mean amount of mouth opening in different time points

Table 2 shows the mean amount of mouth opening in the two groups at different time points. The mean amount of mouth opening was not significantly different between the two groups before or immediately after surgery. However, it was greater in the laser group at 2 (*P* = 0.011) and 7 days (*P* = 0.027), postoperatively (Fig. 3). Repeated measures ANOVA showed that the trend of change in trismus was significant in each group (*P* < 0.05). Although the trend of change in mouth opening was greater in the intervention group, the difference in this respect was not significant between the two groups (*P* = 0.13). The Bonferroni test showed that the mean amount of mouth opening trismus decreased in both groups in the first 2 days and then increased (Table 3).

**Table 2:** Mean amount of mouth opening in the two groups at different time points

Time	Laser		Placebo		*P* value
	Std. deviation	Mean	Std. deviation	Mean	
Before surgery	0.27	5.15	0.34	5.01	0.618
Immediately after surgery	0.18	4.78	0.44	4.59	0.510
After 2 days	0.16	3.94	0.25	3.22	0.011
After 7 days	0.18	4.67	0.14	4.09	0.027

**Table 3:** Pairwise comparisons of trismus at different time points in the two groups

Time (I)	Time (J)	Laser			Placebo		
		Mean difference	Std. deviation	*P* value	Mean difference	Std. deviation	*P* value
Before surgery	Immediately after surgery	0.372^*^	0.114	0.020	0.420^*^	0.140	0.037
	After 2 days	1.216^*^	0.129	0.0001	1.788^*^	0.101	0.0001
	After 7 days	0.480^*^	0.075	0.0001	0.920^*^	0.100	0.0001
Immediately after surgery	After 2 days	0.844^*^	0.120	0.0001	1.368^*^	0.117	0.0001
	After 7 days	0.108	0.138	0.999	0.500^*^	0.170	0.042
After 2 days	After 7 days	− 0.736^*^	0.102	0.0001	− 0.868^*^	0.101	0.0001

* *P* < 0.05

### Pain

**Fig. 4:**
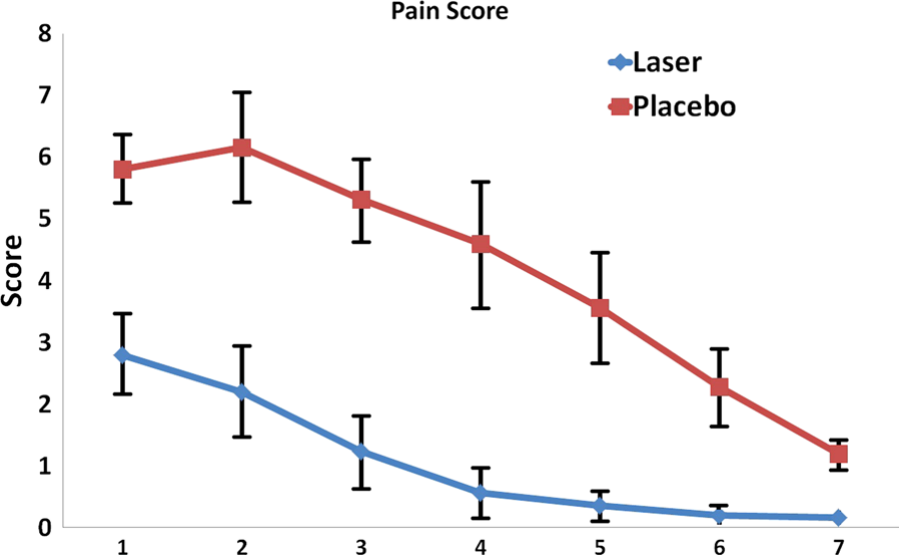
Mean pain score in different time points

Table 4 shows the mean pain score at different time points in the two groups. The mean pain score was significantly higher in the control group in the first 7 days, postoperatively (Fig. 4). Repeated measures ANOVA showed that the trend of change in pain score was descending and significant in each group (*P* < 0.05). The difference in this respect was also significant between the two groups, and the mean change in pain score was lower in the laser group. Table 5 shows pairwise comparisons of pain scores in the two groups at different time points. As shown, the mean pain score decreased over time in the two groups and this reduction was significant in the two groups after the second day, postoperatively.

**Table 4:** Mean pain score at different time points in the two groups

Time (days)	Laser		Placebo		*P* value <
	Std. deviation	Mean	Std. deviation	Mean	
1	0.66	2.8	0.55	5.8	0.05
2	0.74	2.2	0.84	6.16	0.05
3	0.56	1.24	0.64	5.32	0.05
4	0.41	0.56	1.03	4.6	0.05
5	0.22	0.36	0.87	3.56	0.05
6	0.12	0.2	0.61	2.28	0.05
7	0.08	0.16	0.25	1.2	0.05

**Table 5:** Pairwise comparisons of pain scores in the two groups at different time points

Time (I)	Time (J)	Laser			Placebo		
		Mean difference	Std. deviation	*P* value	Mean difference	Std. deviation	*P* value
2	3	0.960^*^	0.179	0.0001	0.840^*^	0.174	0.001
	4	1.640^*^	0.209	0.0001	1.560^*^	0.212	0.0001
	5	1.840^*^	0.203	0.0001	2.600^*^	0.218	0.0001
	6	2.000^*^	0.183	0.0001	3.880^*^	0.308	0.0001
	7	2.040^*^	0.173	0.0001	4.960^*^	0.253	0.0001
3	4	0.680^*^	0.153	0.003	0.720^*^	0.208	0.0321
	5	0.880^*^	0.138	0.0001	1.760^*^	0.157	0.0001
	6	1.040^*^	0.163	0.0001	3.040^*^	0.253	0.0001
	7	1.080^*^	0.166	0.0001	4.120^*^	0.198	0.0001
4	5	0.200	0.090	0.533	1.040^*^	0.137	0.0001
	6	0.360	0.134	0.201	2.320^*^	0.233	0.0001
	7	0.400	0.147	0.184	3.400^*^	0.236	0.0001
5	6	0.160	0.093	0.999	1.280^*^	0.215	0.0001
	7	0.200	0.107	0.999	2.360^*^	0.202	0.0001
6	7	0.040	0.035	0.999	1.080^*^	0.176	0.0001

* *P* < 0.05

## Discussion

This study assessed the effect of extraoral 940 nm low-level diode laser on pain, edema and trismus following surgical extraction of mandibular third molars. The results showed that although pain and edema were lower in the laser group after surgery, this difference was only significant for pain. In a recent study, the authors used 810 nm diode laser with 32 J/cm^2^ energy for pain reduction and reported promising results [26]. However, we used a lower energy density (10 J/cm^2^), different wavelength (940 nm), extraoral irradiation and found the same results. Another study confirmed the optimal efficacy of low-level laser for reduction of pain and edema, postoperatively [22]. However, the authors used 4 J/cm^2^ low-level laser irradiation to the operative side, intraorally and extraorally.

On the contrary, some others did not confirm the efficacy of LLLT for reduction of postoperative pain and edema [21–23, 30]. It should be noted that they used LLLT in multiple sessions while we adopted the single-session protocol. The main difference between our study and that of Eroglu et al. [21] may be the energy density of laser, which was 4 J/cm^2^ in their study and 10 J/cm^2^ in our study. In a recent study and inconsistent with our research, LLLT with 660 nm or 810 nm lasers or their combination was demonstrated to be ineffective for pain relief following tooth extraction [31]. Reduction of pain, swelling, and trismus after the surgical extraction of third molars was also demonstrated using LLLT [24]. Although swelling and trismus were decreased as well as pain, but it was achieved using a combination of intra- and extraoral protocol which takes more time and effort in comparison to extraoral single-session LLLT.

In this study, laser was irradiated extraorally; thus, the epidermis can serve as a barrier against the penetration of laser energy and reaching to deep tissues. This may explain the inefficacy of laser irradiation in our study for resolution of trismus. Another reason may be the fact that the medial pterygoid muscle was not in the field of extraoral laser irradiation, and only the masseter muscle was irradiated. As we know, inflammation of the medial pterygoid muscle plays a major role in trismus, and the masseter muscle has a very small role in the occurrence of trismus. The low energy density of laser may be another reason for its inefficacy in resolution of trismus because the lower the laser energy, the lower its thermal effects on muscle relaxation would be [21, 32]. The energy density of 4 J/cm^2^ is considered as the minimal energy density required for pain relief and elimination of edema, postoperatively [15, 16, 21, 22]. The various administered energy density is one of the main reasons of disagreement between the various findings of the clinical trials investigating the efficacy of LLLT for the reduction of pain, swelling and trismus. In a recent extensive review, Hosseinpour et al. have found that the energy density within 3 and 85.7 J/cm2 is most effective for pain reduction following tooth extraction. Herein, we used the energy density of 10 J/cm^2^ for extraoral single-session LLLT.

LLLT activates the macrophages and regulates the intracapillary pressure by decreasing the vascular permeability, and exerts anti-inflammatory effects as such [30, 33]. In addition, the time of laser therapy and its duration are important parameters affecting the efficacy of LLLT [32]. Inflammatory response is common following surgical procedures. Thus, application of LLLT immediately after surgery and at the onset of the inflammatory process when there is still no edema would be highly effective. The current study showed that single-session irradiation of low-level laser decreased edema in the laser side; although the difference in edema between the laser and placebo sides did not reach statistical significance. In addition, LLLT has been demonstrated to accelerate the process of wound healing by promoting the biostimulation of fibroblasts and enhancing their growth, proliferation, and differentiation as well as pain reduction and inflammation control [34].

Amarillas-Escobar et al. [16] irradiated laser to six extraoral and intraoral points in four sessions and reported optimal results. However, the results were not significant. Multiple sessions of laser therapy are time consuming for both patients and clinicians, and often decrease the patient compliance. In a recent study, Intan et al. used 905-nm LLLT in three sessions following third molar extraction. They showed that LLLT can reduce pain and trismus from day three in patients underwent tooth extraction [35]. Herein, we adopted the single-session protocol and obtained favorable results.

In this study, we adopted the split-mouth design to minimize the effect of demographic factors and inter-individual differences on the results. Some studies have reported that younger patients and males often experience more pain [36, 37]. In this study, no significant difference was noted in pain score between males and females, and the difference in pain score experienced by the oldest and youngest patients was very small. Also, we selected patients with bilaterally impacted third molars with the same level of difficulty (class A according to the Pell and Gregory classification [28]).

The lower the level of pro-inflammatory mediators, the lower the level of pain mediators and consequently the lower the pain score would be. In this split-mouth clinical trial, decision regarding the order of surgical procedures (the first surgical procedure to be the test or the placebo procedure) was made by tossing a coin. Thus, controlling the intensity of pain after the second surgical procedure was impossible because the pain threshold of patients would change after the first surgery [38]. In this study, in almost half the patients, the placebo procedure was performed first while in others, the laser procedure was performed first. However, clinical findings revealed that even in patients who underwent the placebo procedure first, the pain score was lower after the second procedure and laser therapy; this indicates the positive efficacy of LLLT for pain control, which was significant. In general, controversy regarding the efficacy of laser for pain relief, and resolution of edema and trismus may be due to the variations in the study designs, different methods of assessment of pain, edema and trismus, use of different laser types and handpieces, and variations in the laser exposure parameters. Relatively small sample size was a limitation of this study. Future studies with a larger sample size on different laser types and parameters are required.

## Conclusion

Single-session extraoral irradiation of 940 nm diode laser can effectively decrease pain following third molar extraction surgery.

## Data Availability

The datasets used and/or analyzed during the current study are available from the corresponding author on reasonable request.
